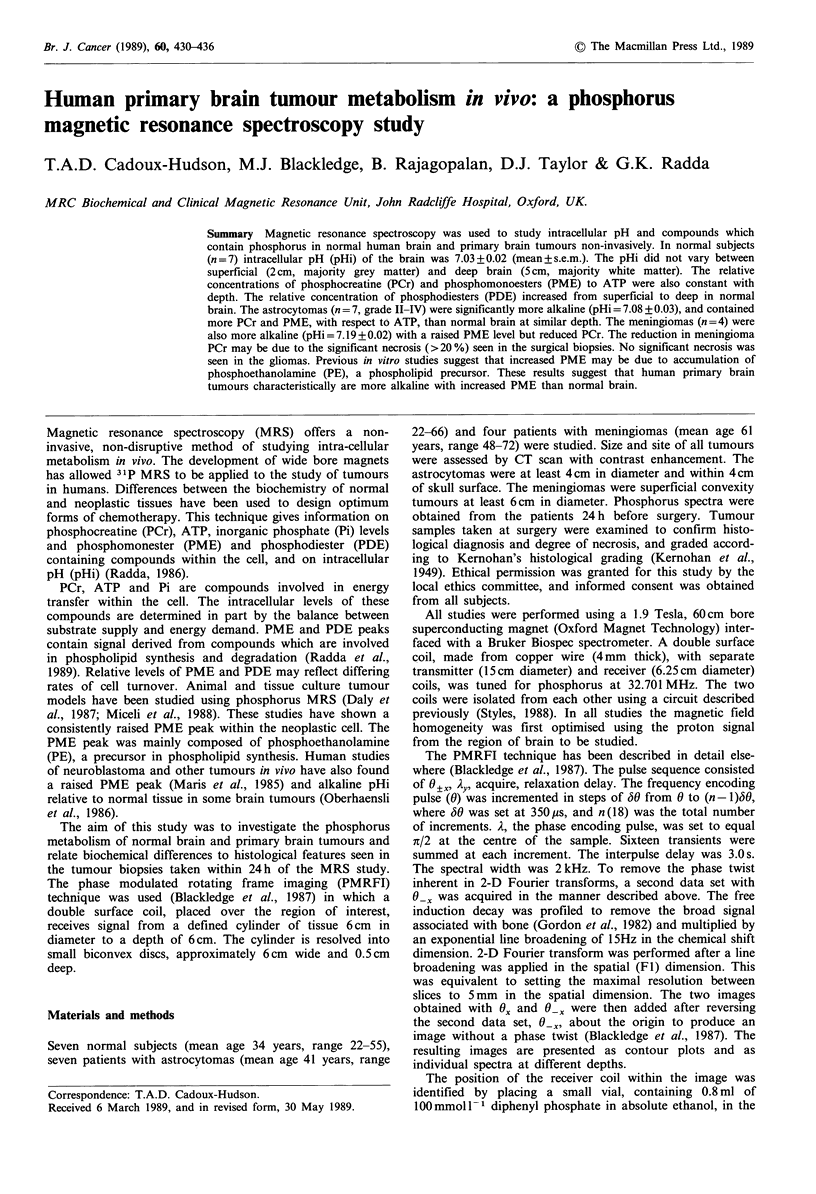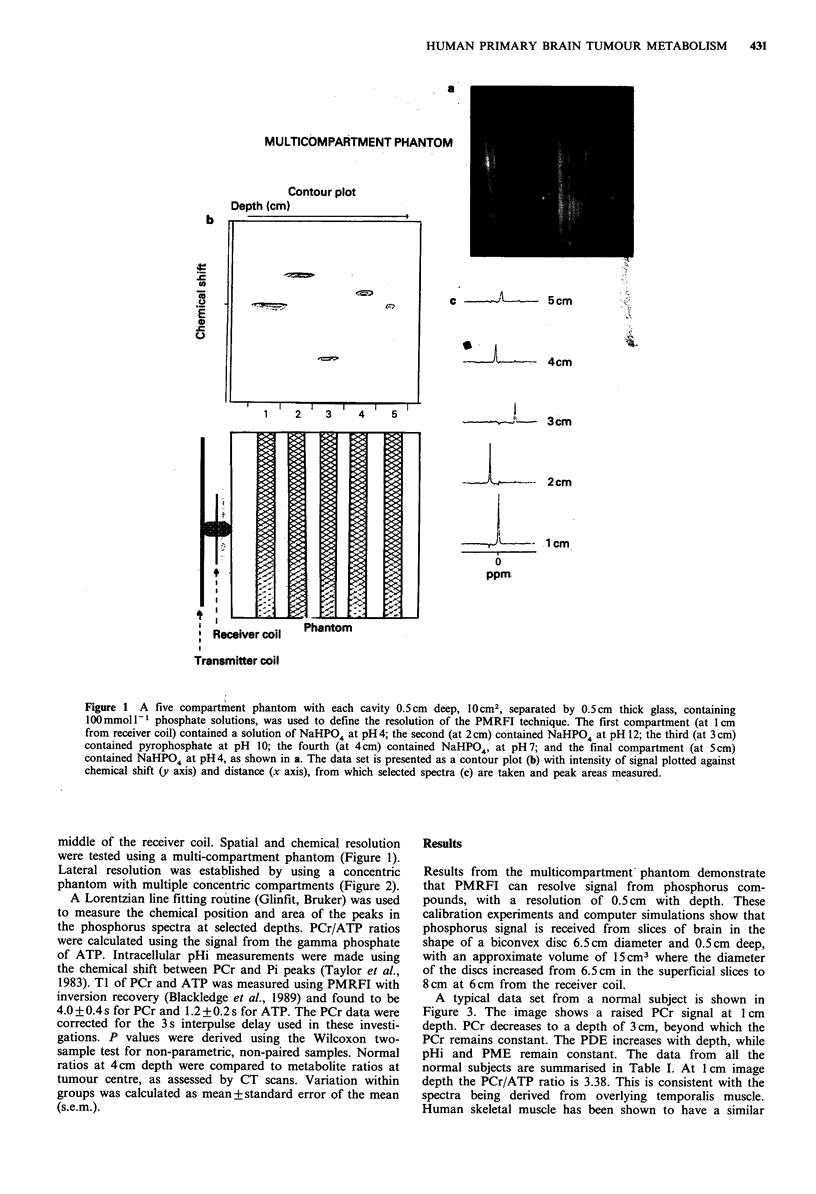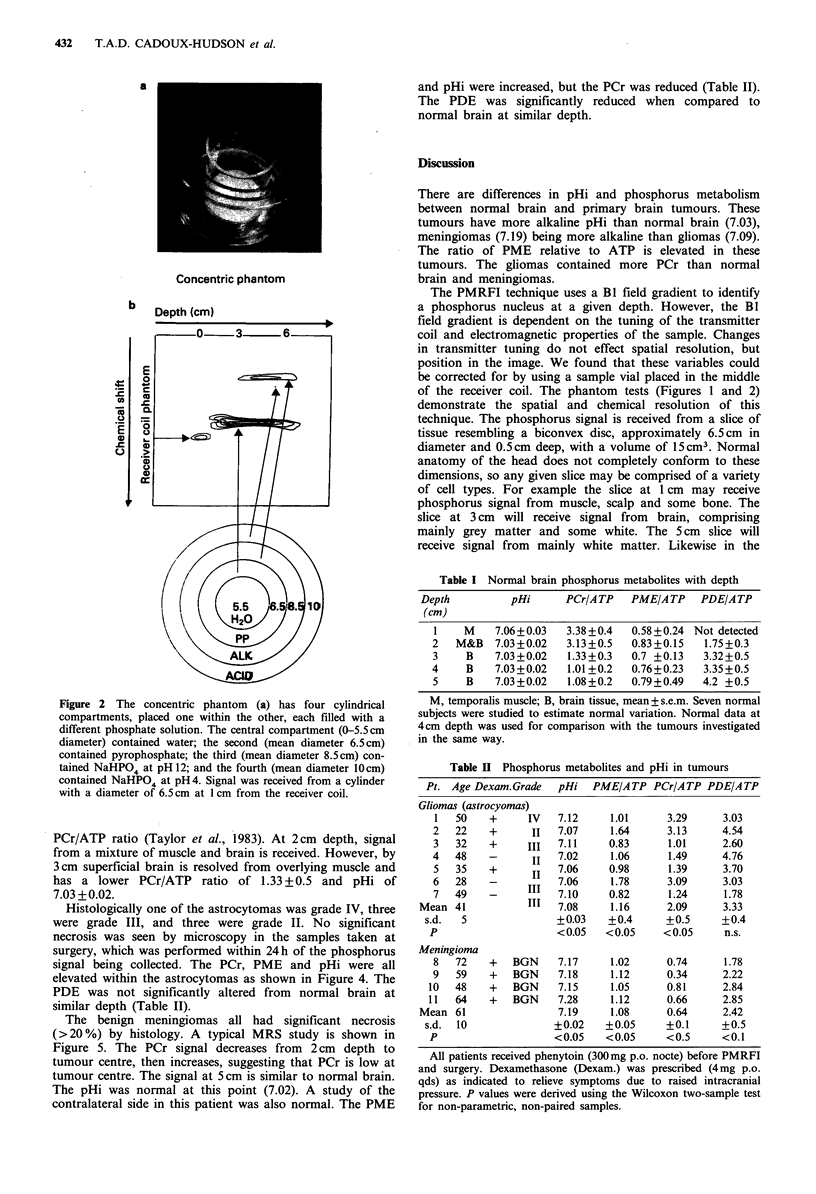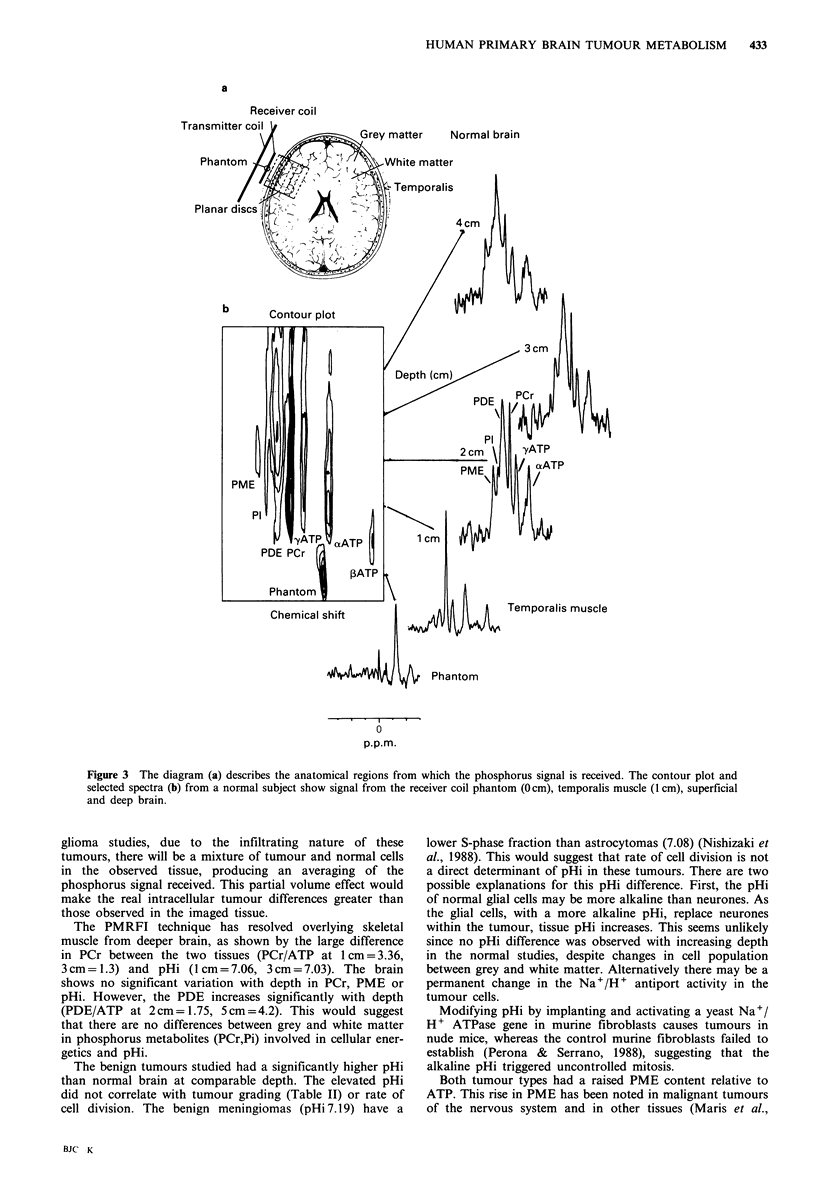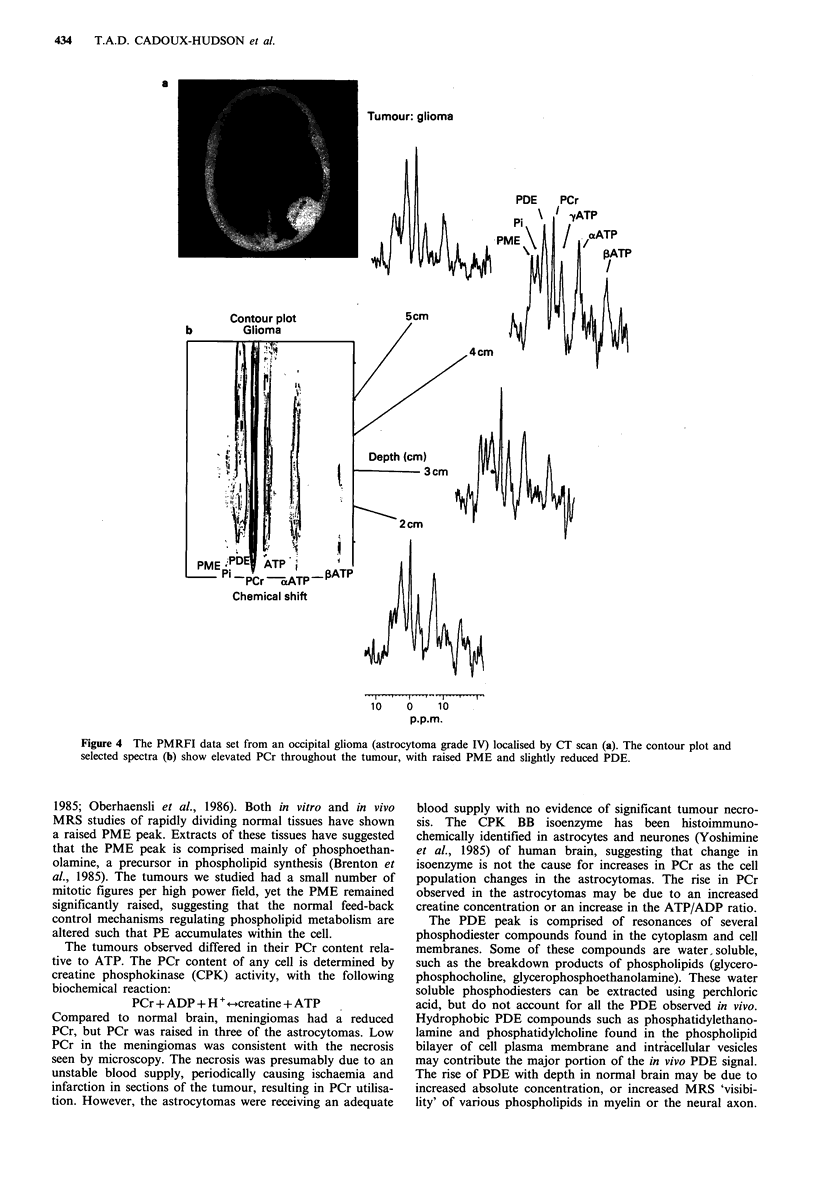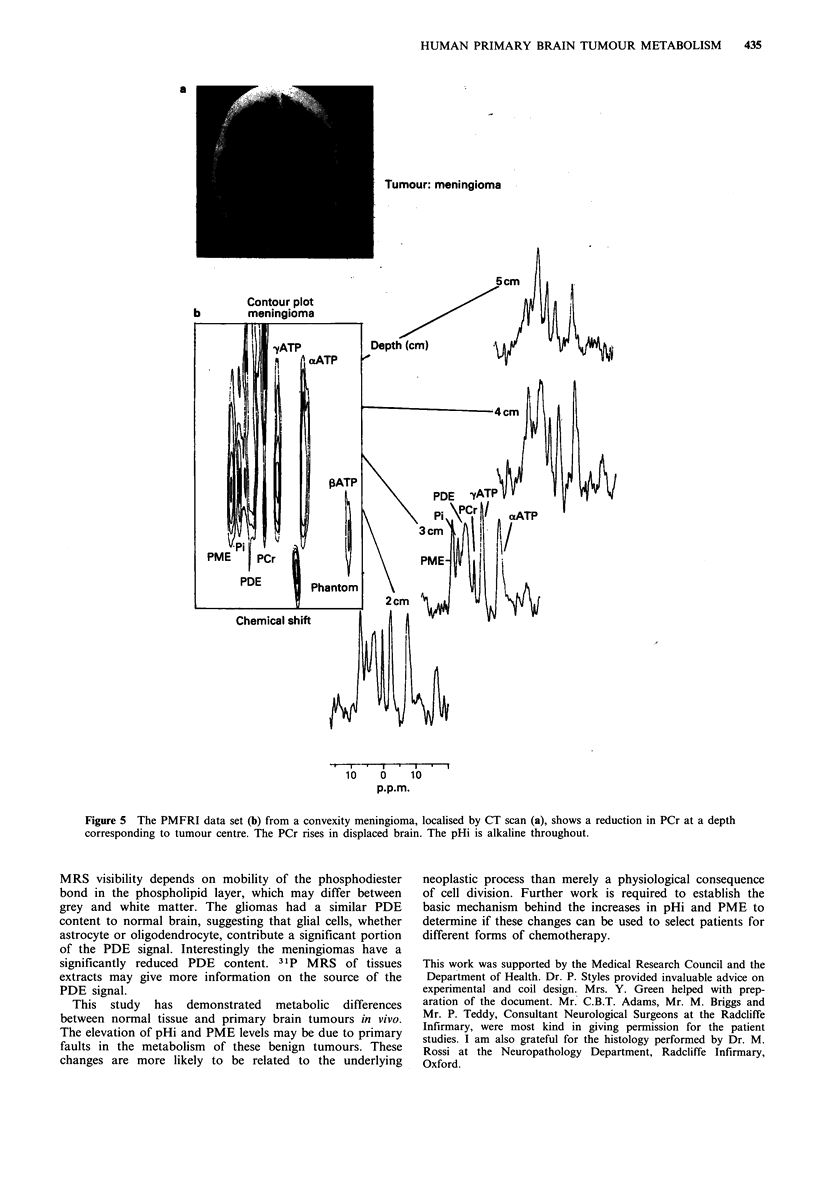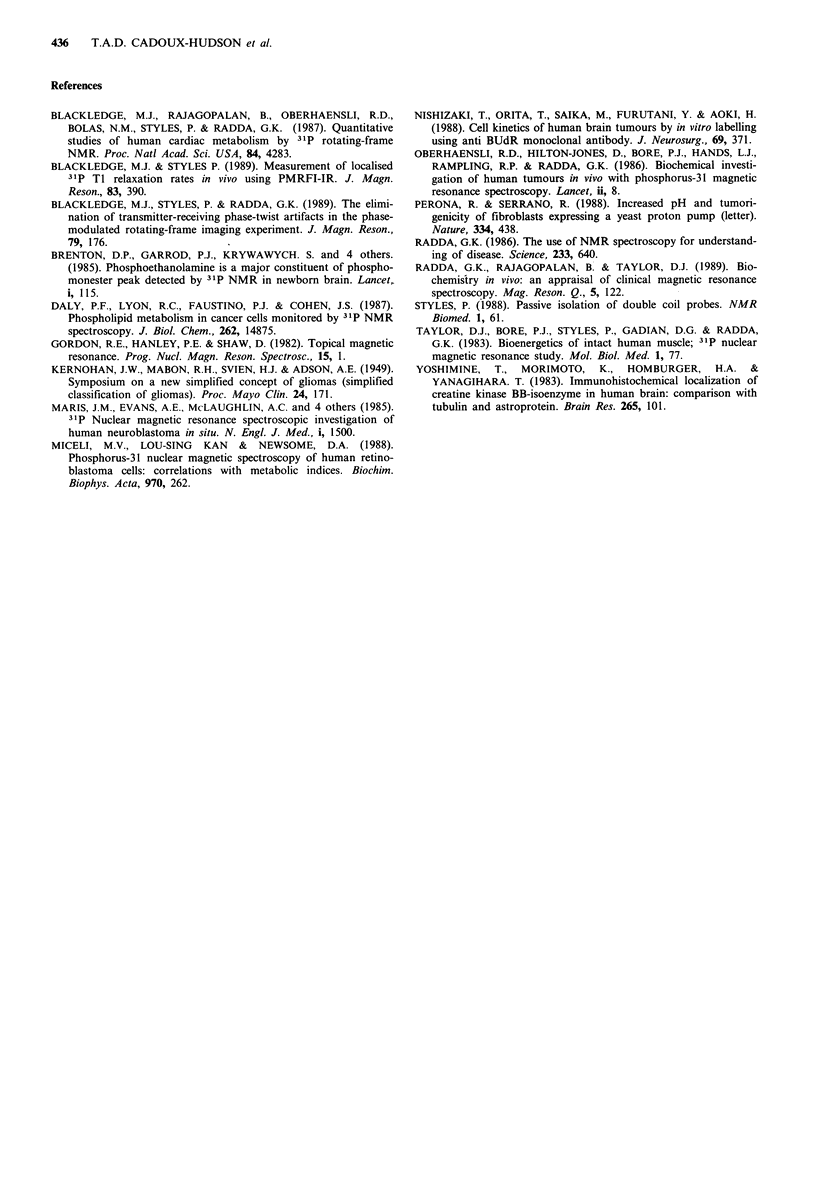# Human primary brain tumour metabolism in vivo: a phosphorus magnetic resonance spectroscopy study.

**DOI:** 10.1038/bjc.1989.300

**Published:** 1989-09

**Authors:** T. A. Cadoux-Hudson, M. J. Blackledge, B. Rajagopalan, D. J. Taylor, G. K. Radda

**Affiliations:** MRC Biochemical and Clinical Magnetic Resonance Unit, John Radcliffe Hospital, Oxford, UK.

## Abstract

**Images:**


 © The Macmillan Press Ltd., 1989

### Human primary brain tumour metabolism *in vivo*: a phosphorus magnetic resonance spectroscopy study

T.A.D. Cadoux-Hudson, M.J. Blackledge, B. Rajagopalan, D.J. Taylor & G.K. Radda

*MRC Biochemical and Clinical Magnetic Resonance Unit, John Radcliffe Hospital, Oxford, UK.*

**Summary** Magnetic resonance spectroscopy was used to study intracellular pH and compounds which contain phosphorus in normal human brain and primary brain tumours non-invasively. In normal subjects ($n=7$) intracellular pH (pHi) of the brain was $7.03 \pm 0.02$ (mean $\pm$ s.e.m.). The pHi did not vary between superficial (2 cm, majority grey matter) and deep brain (5 cm, majority white matter). The relative concentrations of phosphocreatine (PCr) and phosphomonoesters (PME) to ATP were also constant with depth. The relative concentration of phosphodiesters (PDE) increased from superficial to deep in normal brain. The astrocytomas ($n=7$, grade II–IV) were significantly more alkaline (pHi $= 7.08 \pm 0.03$), and contained more PCr and PME, with respect to ATP, than normal brain at similar depth. The meningiomas ($n=4$) were also more alkaline (pHi $= 7.19 \pm 0.02$) with a raised PME level but reduced PCr. The reduction in meningioma PCr may be due to the significant necrosis (>20%) seen in the surgical biopsies. No significant necrosis was seen in the gliomas. Previous *in vitro* studies suggest that increased PME may be due to accumulation of phosphoethanolamine (PE), a phospholipid precursor. These results suggest that human primary brain tumours characteristically are more alkaline with increased PME than normal brain.

Magnetic resonance spectroscopy (MRS) offers a non-invasive, non-disruptive method of studying intra-cellular metabolism *in vivo*. The development of wide bore magnets has allowed $^{31}P$ MRS to be applied to the study of tumours in humans. Differences between the biochemistry of normal and neoplastic tissues have been used to design optimum forms of chemotherapy. This technique gives information on phosphocreatine (PCr), ATP, inorganic phosphate (Pi) levels and phosphomonester (PME) and phosphodiester (PDE) containing compounds within the cell, and on intracellular pH (pHi) (Radda, 1986).

PCr, ATP and Pi are compounds involved in energy transfer within the cell. The intracellular levels of these compounds are determined in part by the balance between substrate supply and energy demand. PME and PDE peaks contain signal derived from compounds which are involved in phospholipid synthesis and degradation (Radda *et al.*, 1989). Relative levels of PME and PDE may reflect differing rates of cell turnover. Animal and tissue culture tumour models have been studied using phosphorus MRS (Daly *et al.*, 1987; Miceli *et al.*, 1988). These studies have shown a consistently raised PME peak within the neoplastic cell. The PME peak was mainly composed of phosphoethanolamine (PE), a precursor in phospholipid synthesis. Human studies of neuroblastoma and other tumours *in vivo* have also found a raised PME peak (Maris *et al.*, 1985) and alkaline pHi relative to normal tissue in some brain tumours (Oberhaensli *et al.*, 1986).

The aim of this study was to investigate the phosphorus metabolism of normal brain and primary brain tumours and relate biochemical differences to histological features seen in the tumour biopsies taken within 24 h of the MRS study. The phase modulated rotating frame imaging (PMRFI) technique was used (Blackledge *et al.*, 1987) in which a double surface coil, placed over the region of interest, receives signal from a defined cylinder of tissue 6 cm in diameter to a depth of 6 cm. The cylinder is resolved into small biconvex discs, approximately 6 cm wide and 0.5 cm deep.

### Materials and methods

Seven normal subjects (mean age 34 years, range 22–55), seven patients with astrocytomas (mean age 41 years, range 22–66) and four patients with meningiomas (mean age 61 years, range 48–72) were studied. Size and site of all tumours were assessed by CT scan with contrast enhancement. The astrocytomas were at least 4 cm in diameter and within 4 cm of skull surface. The meningiomas were superficial convexity tumours at least 6 cm in diameter. Phosphorus spectra were obtained from the patients 24 h before surgery. Tumour samples taken at surgery were examined to confirm histological diagnosis and degree of necrosis, and graded according to Kernohan's histological grading (Kernohan *et al.*, 1949). Ethical permission was granted for this study by the local ethics committee, and informed consent was obtained from all subjects.

All studies were performed using a 1.9 Tesla, 60 cm bore superconducting magnet (Oxford Magnet Technology) interfaced with a Bruker Biospec spectrometer. A double surface coil, made from copper wire (4 mm thick), with separate transmitter (15 cm diameter) and receiver (6.25 cm diameter) coils, was tuned for phosphorus at 32.701 MHz. The two coils were isolated from each other using a circuit described previously (Styles, 1988). In all studies the magnetic field homogeneity was first optimised using the proton signal from the region of brain to be studied.

The PMRFI technique has been described in detail elsewhere (Blackledge *et al.*, 1987). The pulse sequence consisted of $\theta_{\pm x}$, $\lambda_y$, acquire, relaxation delay. The frequency encoding pulse ($\theta$) was incremented in steps of $\delta\theta$ from $\theta$ to $(n-1)\delta\theta$, where $\delta\theta$ was set at 350 $\mu$s, and $n$(18) was the total number of increments. $\lambda$, the phase encoding pulse, was set to equal $\pi/2$ at the centre of the sample. Sixteen transients were summed at each increment. The interpulse delay was 3.0 s. The spectral width was 2 kHz. To remove the phase twist inherent in 2-D Fourier transforms, a second data set with $\theta_{-x}$ was acquired in the manner described above. The free induction decay was profiled to remove the broad signal associated with bone (Gordon *et al.*, 1982) and multiplied by an exponential line broadening of 15Hz in the chemical shift dimension. 2-D Fourier transform was performed after a line broadening was applied in the spatial (F1) dimension. This was equivalent to setting the maximal resolution between slices to 5 mm in the spatial dimension. The two images obtained with $\theta_x$ and $\theta_{-x}$ were then added after reversing the second data set, $\theta_{-x}$, about the origin to produce an image without a phase twist (Blackledge *et al.*, 1987). The resulting images are presented as contour plots and as individual spectra at different depths.

The position of the receiver coil within the image was identified by placing a small vial, containing 0.8 ml of 100 mmol l$^{-1}$ diphenyl phosphate in absolute ethanol, in the

Correspondence: T.A.D. Cadoux-Hudson.
Received 6 March 1989, and in revised form, 30 May 1989.

**Figure 1** A five compartment phantom with each cavity 0.5 cm deep, 10 cm$^2$, separated by 0.5 cm thick glass, containing 100 mmol l$^{-1}$ phosphate solutions, was used to define the resolution of the PMRFI technique. The first compartment (at 1 cm from receiver coil) contained a solution of $NaHPO_4$ at pH 4; the second (at 2 cm) contained $NaHPO_4$ at pH 12; the third (at 3 cm) contained pyrophosphate at pH 10; the fourth (at 4 cm) contained $NaHPO_4$, at pH 7; and the final compartment (at 5 cm) contained $NaHPO_4$ at pH 4, as shown in **a**. The data set is presented as a contour plot (**b**) with intensity of signal plotted against chemical shift ($y$ axis) and distance ($x$ axis), from which selected spectra (**c**) are taken and peak areas measured.

middle of the receiver coil. Spatial and chemical resolution were tested using a multi-compartment phantom (Figure 1). Lateral resolution was established by using a concentric phantom with multiple concentric compartments (Figure 2).

A Lorentzian line fitting routine (Glinfit, Bruker) was used to measure the chemical position and area of the peaks in the phosphorus spectra at selected depths. PCr/ATP ratios were calculated using the signal from the gamma phosphate of ATP. Intracellular pHi measurements were made using the chemical shift between PCr and Pi peaks (Taylor *et al.*, 1983). T1 of PCr and ATP was measured using PMRFI with inversion recovery (Blackledge *et al.*, 1989) and found to be $4.0 \pm 0.4$ s for PCr and $1.2 \pm 0.2$ s for ATP. The PCr data were corrected for the 3 s interpulse delay used in these investigations. *P* values were derived using the Wilcoxon two-sample test for non-parametric, non-paired samples. Normal ratios at 4 cm depth were compared to metabolite ratios at tumour centre, as assessed by CT scans. Variation within groups was calculated as mean $\pm$ standard error of the mean (s.e.m.).

### Results

Results from the multicompartment phantom demonstrate that PMRFI can resolve signal from phosphorus compounds, with a resolution of 0.5 cm with depth. These calibration experiments and computer simulations show that phosphorus signal is received from slices of brain in the shape of a biconvex disc 6.5 cm diameter and 0.5 cm deep, with an approximate volume of 15 cm$^3$ where the diameter of the discs increased from 6.5 cm in the superficial slices to 8 cm at 6 cm from the receiver coil.

A typical data set from a normal subject is shown in Figure 3. The image shows a raised PCr signal at 1 cm depth. PCr decreases to a depth of 3 cm, beyond which the PCr remains constant. The PDE increases with depth, while pHi and PME remain constant. The data from all the normal subjects are summarised in Table I. At 1 cm image depth the PCr/ATP ratio is 3.38. This is consistent with the spectra being derived from overlying temporalis muscle. Human skeletal muscle has been shown to have a similar

**Figure 2** The concentric phantom (**a**) has four cylindrical compartments, placed one within the other, each filled with a different phosphate solution. The central compartment (0–5.5 cm diameter) contained water; the second (mean diameter 6.5 cm) contained pyrophosphate; the third (mean diameter 8.5 cm) contained $NaHPO_4$ at pH 12; and the fourth (mean diameter 10 cm) contained $NaHPO_4$ at pH 4. Signal was received from a cylinder with a diameter of 6.5 cm at 1 cm from the receiver coil.

PCr/ATP ratio (Taylor *et al.*, 1983). At 2 cm depth, signal from a mixture of muscle and brain is received. However, by 3 cm superficial brain is resolved from overlying muscle and has a lower PCr/ATP ratio of 1.33±0.5 and pHi of 7.03±0.02.

Histologically one of the astrocytomas was grade IV, three were grade III, and three were grade II. No significant necrosis was seen by microscopy in the samples taken at surgery, which was performed within 24 h of the phosphorus signal being collected. The PCr, PME and pHi were all elevated within the astrocytomas as shown in Figure 4. The PDE was not significantly altered from normal brain at similar depth (Table II).

The benign meningiomas all had significant necrosis (>20%) by histology. A typical MRS study is shown in Figure 5. The PCr signal decreases from 2 cm depth to tumour centre, then increases, suggesting that PCr is low at tumour centre. The signal at 5 cm is similar to normal brain. The pHi was normal at this point (7.02). A study of the contralateral side in this patient was also normal. The PME and pHi were increased, but the PCr was reduced (Table II). The PDE was significantly reduced when compared to normal brain at similar depth.

### Discussion

There are differences in pHi and phosphorus metabolism between normal brain and primary brain tumours. These tumours have more alkaline pHi than normal brain (7.03), meningiomas (7.19) being more alkaline than gliomas (7.09). The ratio of PME relative to ATP is elevated in these tumours. The gliomas contained more PCr than normal brain and meningiomas.

The PMRFI technique uses a B1 field gradient to identify a phosphorus nucleus at a given depth. However, the B1 field gradient is dependent on the tuning of the transmitter coil and electromagnetic properties of the sample. Changes in transmitter tuning do not effect spatial resolution, but position in the image. We found that these variables could be corrected for by using a sample vial placed in the middle of the receiver coil. The phantom tests (Figures 1 and 2) demonstrate the spatial and chemical resolution of this technique. The phosphorus signal is received from a slice of tissue resembling a biconvex disc, approximately 6.5 cm in diameter and 0.5 cm deep, with a volume of 15 $cm^3$. Normal anatomy of the head does not completely conform to these dimensions, so any given slice may be comprised of a variety of cell types. For example the slice at 1 cm may receive phosphorus signal from muscle, scalp and some bone. The slice at 3 cm will receive signal from brain, comprising mainly grey matter and some white. The 5 cm slice will receive signal from mainly white matter. Likewise in the

**Table I** Normal brain phosphorus metabolites with depth

| *Depth (cm)* | | *pHi* | *PCr/ATP* | *PME/ATP* | *PDE/ATP* |
|---|---|---|---|---|---|
| 1 | M | 7.06±0.03 | 3.38±0.4 | 0.58±0.24 | Not detected |
| 2 | M&B | 7.03±0.02 | 3.13±0.5 | 0.83±0.15 | 1.75±0.3 |
| 3 | B | 7.03±0.02 | 1.33±0.3 | 0.7 ±0.13 | 3.32±0.5 |
| 4 | B | 7.03±0.02 | 1.01±0.2 | 0.76±0.23 | 3.35±0.5 |
| 5 | B | 7.03±0.02 | 1.08±0.2 | 0.79±0.49 | 4.2 ±0.5 |

M, temporalis muscle; B, brain tissue, mean±s.e.m. Seven normal subjects were studied to estimate normal variation. Normal data at 4 cm depth was used for comparison with the tumours investigated in the same way.

**Table II** Phosphorus metabolites and pHi in tumours

| *Pt.* | *Age* | *Dexam.* | *Grade* | *pHi* | *PME/ATP* | *PCr/ATP* | *PDE/ATP* |
|---|---|---|---|---|---|---|---|
| *Gliomas (astrocyomas)* | | | | | | | |
| 1 | 50 | + | IV | 7.12 | 1.01 | 3.29 | 3.03 |
| 2 | 22 | + | II | 7.07 | 1.64 | 3.13 | 4.54 |
| 3 | 32 | + | III | 7.11 | 0.83 | 1.01 | 2.60 |
| 4 | 48 | – | II | 7.02 | 1.06 | 1.49 | 4.76 |
| 5 | 35 | + | II | 7.06 | 0.98 | 1.39 | 3.70 |
| 6 | 28 | – | III | 7.06 | 1.78 | 3.09 | 3.03 |
| 7 | 49 | – | III | 7.10 | 0.82 | 1.24 | 1.78 |
| Mean | 41 | | | 7.08 | 1.16 | 2.09 | 3.33 |
| s.d. | 5 | | | ±0.03 | ±0.4 | ±0.5 | ±0.4 |
| *P* | | | | <0.05 | <0.05 | <0.05 | n.s. |
| *Meningioma* | | | | | | | |
| 8 | 72 | + | BGN | 7.17 | 1.02 | 0.74 | 1.78 |
| 9 | 59 | + | BGN | 7.18 | 1.12 | 0.34 | 2.22 |
| 10 | 48 | + | BGN | 7.15 | 1.05 | 0.81 | 2.84 |
| 11 | 64 | + | BGN | 7.28 | 1.12 | 0.66 | 2.85 |
| Mean | 61 | | | 7.19 | 1.08 | 0.64 | 2.42 |
| s.d. | 10 | | | ±0.02 | ±0.05 | ±0.1 | ±0.5 |
| *P* | | | | <0.05 | <0.05 | <0.5 | <0.1 |

All patients received phenytoin (300 mg p.o. nocte) before PMRFI and surgery. Dexamethasone (Dexam.) was prescribed (4 mg p.o. qds) as indicated to relieve symptoms due to raised intracranial pressure. *P* values were derived using the Wilcoxon two-sample test for non-parametric, non-paired samples.

**Figure 3** The diagram (**a**) describes the anatomical regions from which the phosphorus signal is received. The contour plot and selected spectra (**b**) from a normal subject show signal from the receiver coil phantom (0 cm), temporalis muscle (1 cm), superficial and deep brain.

glioma studies, due to the infiltrating nature of these tumours, there will be a mixture of tumour and normal cells in the observed tissue, producing an averaging of the phosphorus signal received. This partial volume effect would make the real intracellular tumour differences greater than those observed in the imaged tissue.

The PMRFI technique has resolved overlying skeletal muscle from deeper brain, as shown by the large difference in PCr between the two tissues (PCr/ATP at 1 cm = 3.36, 3 cm = 1.3) and pHi (1 cm = 7.06, 3 cm = 7.03). The brain shows no significant variation with depth in PCr, PME or pHi. However, the PDE increases significantly with depth (PDE/ATP at 2 cm = 1.75, 5 cm = 4.2). This would suggest that there are no differences between grey and white matter in phosphorus metabolites (PCr,Pi) involved in cellular energetics and pHi.

The benign tumours studied had a significantly higher pHi than normal brain at comparable depth. The elevated pHi did not correlate with tumour grading (Table II) or rate of cell division. The benign meningiomas (pHi 7.19) have a lower S-phase fraction than astrocytomas (7.08) (Nishizaki *et al.*, 1988). This would suggest that rate of cell division is not a direct determinant of pHi in these tumours. There are two possible explanations for this pHi difference. First, the pHi of normal glial cells may be more alkaline than neurones. As the glial cells, with a more alkaline pHi, replace neurones within the tumour, tissue pHi increases. This seems unlikely since no pHi difference was observed with increasing depth in the normal studies, despite changes in cell population between grey and white matter. Alternatively there may be a permanent change in the $Na^+/H^+$ antiport activity in the tumour cells.

Modifying pHi by implanting and activating a yeast $Na^+/H^+$ ATPase gene in murine fibroblasts causes tumours in nude mice, whereas the control murine fibroblasts failed to establish (Perona & Serrano, 1988), suggesting that the alkaline pHi triggered uncontrolled mitosis.

Both tumour types had a raised PME content relative to ATP. This rise in PME has been noted in malignant tumours of the nervous system and in other tissues (Maris *et al.*,

**Figure 4** The PMRFI data set from an occipital glioma (astrocytoma grade IV) localised by CT scan (**a**). The contour plot and selected spectra (**b**) show elevated PCr throughout the tumour, with raised PME and slightly reduced PDE.

1985; Oberhaensli *et al.*, 1986). Both *in vitro* and *in vivo* MRS studies of rapidly dividing normal tissues have shown a raised PME peak. Extracts of these tissues have suggested that the PME peak is comprised mainly of phosphoethanolamine, a precursor in phospholipid synthesis (Brenton *et al.*, 1985). The tumours we studied had a small number of mitotic figures per high power field, yet the PME remained significantly raised, suggesting that the normal feed-back control mechanisms regulating phospholipid metabolism are altered such that PE accumulates within the cell.

The tumours observed differed in their PCr content relative to ATP. The PCr content of any cell is determined by creatine phosphokinase (CPK) activity, with the following biochemical reaction:

$$PCr + ADP + H^{+} \leftrightarrow creatine + ATP$$

Compared to normal brain, meningiomas had a reduced PCr, but PCr was raised in three of the astrocytomas. Low PCr in the meningiomas was consistent with the necrosis seen by microscopy. The necrosis was presumably due to an unstable blood supply, periodically causing ischaemia and infarction in sections of the tumour, resulting in PCr utilisation. However, the astrocytomas were receiving an adequate blood supply with no evidence of significant tumour necrosis. The CPK BB isoenzyme has been histoimmunochemically identified in astrocytes and neurones (Yoshimine *et al.*, 1985) of human brain, suggesting that change in isoenzyme is not the cause for increases in PCr as the cell population changes in the astrocytomas. The rise in PCr observed in the astrocytomas may be due to an increased creatine concentration or an increase in the ATP/ADP ratio.

The PDE peak is comprised of resonances of several phosphodiester compounds found in the cytoplasm and cell membranes. Some of these compounds are water-soluble, such as the breakdown products of phospholipids (glycerophosphocholine, glycerophosphoethanolamine). These water soluble phosphodiesters can be extracted using perchloric acid, but do not account for all the PDE observed *in vivo*. Hydrophobic PDE compounds such as phosphatidylethanolamine and phosphatidylcholine found in the phospholipid bilayer of cell plasma membrane and intracellular vesicles may contribute the major portion of the *in vivo* PDE signal. The rise of PDE with depth in normal brain may be due to increased absolute concentration, or increased MRS 'visibility' of various phospholipids in myelin or the neural axon.

**Figure 5** The PMFRI data set (**b**) from a convexity meningioma, localised by CT scan (**a**), shows a reduction in PCr at a depth corresponding to tumour centre. The PCr rises in displaced brain. The pHi is alkaline throughout.

MRS visibility depends on mobility of the phosphodiester bond in the phospholipid layer, which may differ between grey and white matter. The gliomas had a similar PDE content to normal brain, suggesting that glial cells, whether astrocyte or oligodendrocyte, contribute a significant portion of the PDE signal. Interestingly the meningiomas have a significantly reduced PDE content. $^{31}P$ MRS of tissues extracts may give more information on the source of the PDE signal.

This study has demonstrated metabolic differences between normal tissue and primary brain tumours *in vivo*. The elevation of pHi and PME levels may be due to primary faults in the metabolism of these benign tumours. These changes are more likely to be related to the underlying neoplastic process than merely a physiological consequence of cell division. Further work is required to establish the basic mechanism behind the increases in pHi and PME to determine if these changes can be used to select patients for different forms of chemotherapy.

This work was supported by the Medical Research Council and the Department of Health. Dr. P. Styles provided invaluable advice on experimental and coil design. Mrs. Y. Green helped with preparation of the document. Mr. C.B.T. Adams, Mr. M. Briggs and Mr. P. Teddy, Consultant Neurological Surgeons at the Radcliffe Infirmary, were most kind in giving permission for the patient studies. I am also grateful for the histology performed by Dr. M. Rossi at the Neuropathology Department, Radcliffe Infirmary, Oxford.

### References

BLACKLEDGE, M.J., RAJAGOPALAN, B., OBERHAENSLI, R.D., BOLAS, N.M., STYLES, P. & RADDA, G.K. (1987). Quantitative studies of human cardiac metabolism by $^{31}P$ rotating-frame NMR. *Proc. Natl Acad. Sci. USA*, **84**, 4283.

BLACKLEDGE, M.J. & STYLES P. (1989). Measurement of localised $^{31}P$ T1 relaxation rates *in vivo* using PMRFI-IR. *J. Magn. Reson.*, **83**, 390.

BLACKLEDGE, M.J., STYLES, P. & RADDA, G.K. (1989). The elimination of transmitter-receiving phase-twist artifacts in the phase-modulated rotating-frame imaging experiment. *J. Magn. Reson.*, **79**, 176.

BRENTON, D.P., GARROD, P.J., KRYWAWYCH. S. and 4 others. (1985). Phosphoethanolamine is a major constituent of phosphomonester peak detected by $^{31}P$ NMR in newborn brain. *Lancet*, **i**, 115.

DALY, P.F., LYON, R.C., FAUSTINO, P.J. & COHEN, J.S. (1987). Phospholipid metabolism in cancer cells monitored by $^{31}P$ NMR spectroscopy. *J. Biol. Chem.*, **262**, 14875.

GORDON, R.E., HANLEY, P.E. & SHAW, D. (1982). Topical magnetic resonance. *Prog. Nucl. Magn. Reson. Spectrosc.*, **15**, 1.

KERNOHAN, J.W., MABON, R.H., SVIEN, H.J. & ADSON, A.E. (1949). Symposium on a new simplified concept of gliomas (simplified classification of gliomas). *Proc. Mayo Clin.* **24**, 171.

MARIS, J.M., EVANS, A.E., McLAUGHLIN, A.C. and 4 others (1985). $^{31}P$ Nuclear magnetic resonance spectroscopic investigation of human neuroblastoma *in situ*. *N. Engl. J. Med.*, **i**, 1500.

MICELI, M.V., LOU-SING KAN & NEWSOME, D.A. (1988). Phosphorus-31 nuclear magnetic spectroscopy of human retinoblastoma cells: correlations with metabolic indices. *Biochim. Biophys. Acta*, **970**, 262.

NISHIZAKI, T., ORITA, T., SAIKA, M., FURUTANI, Y. & AOKI, H. (1988). Cell kinetics of human brain tumours by *in vitro* labelling using anti BUdR monoclonal antibody. *J. Neurosurg.*, **69**, 371.

OBERHAENSLI, R.D., HILTON-JONES, D., BORE, P.J., HANDS, L.J., RAMPLING, R.P. & RADDA, G.K. (1986). Biochemical investigation of human tumours *in vivo* with phosphorus-31 magnetic resonance spectroscopy. *Lancet*, **ii**, 8.

PERONA, R. & SERRANO, R. (1988). Increased pH and tumorigenicity of fibroblasts expressing a yeast proton pump (letter). *Nature*, **334**, 438.

RADDA, G.K. (1986). The use of NMR spectroscopy for understanding of disease. *Science*, **233**, 640.

RADDA, G.K., RAJAGOPALAN, B. & TAYLOR, D.J. (1989). Biochemistry *in vivo*: an appraisal of clinical magnetic resonance spectroscopy. *Mag. Reson. Q.*, **5**, 122.

STYLES, P. (1988). Passive isolation of double coil probes. *NMR Biomed.* **1**, 61.

TAYLOR, D.J., BORE, P.J., STYLES, P., GADIAN, D.G. & RADDA, G.K. (1983). Bioenergetics of intact human muscle; $^{31}P$ nuclear magnetic resonance study. *Mol. Biol. Med.* **1**, 77.

YOSHIMINE, T., MORIMOTO, K., HOMBURGER, H.A. & YANAGIHARA. T. (1983). Immunohistochemical localization of creatine kinase BB-isoenzyme in human brain: comparison with tubulin and astroprotein. *Brain Res.* **265**, 101.